# Malignant teratoid tumor of the thyroid gland: an aggressive primitive multiphenotypic malignancy showing organotypical elements and frequent *DICER1* alterations—is the term “thyroblastoma” more appropriate?

**DOI:** 10.1007/s00428-020-02853-1

**Published:** 2020-06-07

**Authors:** Abbas Agaimy, Leora Witkowski, Robert Stoehr, Joseph Christopher Castillo Cuenca, Carlos Alberto González-Muller, Alfred Brütting, Markus Bährle, Konstantinos Mantsopoulos, Randa M. S. Amin, Arndt Hartmann, Markus Metzler, Samir S. Amr, William D. Foulkes, Manuel Sobrinho-Simões, Catarina Eloy

**Affiliations:** 1 Institute of Pathology, University Hospital Erlangen, Erlangen, Germany; 2 Departments of Human Genetics, McGill University, Montreal, Quebec Canada; 3 Instituto de Anatomía Patológica Arias Stella, Lima, Peru; 4 Department of Surgery, Malteser Waldkrankenhaus, Erlangen, Germany; 5 Department of Otorhinolaryngology, Head and Neck Surgery, University of Erlangen–Nuremberg, Erlangen, Germany; 6 Department of Pathology, King Fahad Specialist Hospital, Dammam, Saudi Arabia; 7 Department of Pediatrics, University Hospital Erlangen, Erlangen, Germany; 8 Cancer Research Program, Research Institute of the McGill University Health Centre, McGill University, Montreal, Quebec Canada; 9 Cancer Genetics Laboratory, Lady Davis Institute, Jewish General Hospital, McGill University Montreal, Montreal, Quebec Canada; 10 Instituto de Investigação e Inovação em Saúde, Porto, Portugal; 11 Institute of Molecular Pathology and Immunology, University of Porto, Porto, Portugal; 12 Medical Faculty, University of Porto, Porto, Portugal; 13 Department of Pathology, Centro Hospitalar S. João, Porto, Portugal

**Keywords:** Malignant teratoma, Teratocarcinosarcoma, Thyroid, Head and neck, Rhabdomyosarcoma, Germ cell tumor, DICER1, Thyroblastoma

## Abstract

Primary thyroid teratomas are exceedingly rare. Mature and immature variants recapitulate their gonadal counterparts (predilection for infants/children, triphasic germ layer differentiation, and favorable outcome). On the other hand, the so-called malignant teratomas affect predominantly adults and elderly, are highly aggressive, and, according to a few published cases, harbor *DICER1* mutations. We describe three highly aggressive sporadic malignant teratoid thyroid tumors in 2 females (17 and 45 years) and one male (17 years). Histology showed triphasic neoplasms composed of solid nests of small primitive monomorphic cells embedded in a cellular stroma with primitive immature rhabdomyosarcoma-like (2) or pleomorphic sarcoma-like (1) phenotype. The third component was represented by TTF1+/PAX8+ primitive teratoid epithelial tubules reminiscent of primitive thyroid follicles and/or Wilms tumor, admixed with scattered respiratory- or enteric-type tubules, neuroepithelial rosettes, and fetal-type squamoid nests. Foci of cartilage were seen in two cases, but none contained mature organoid adult-type tissue or skin adnexa. SALL4 was expressed in the small cell (2) and stromal (1) component. Other germ cell markers were negative. Molecular testing revealed a known “hotspot” pathogenic *DICER1* mutation in two cases. In addition, case 1 had a missense *TP53* variant. This type of thyroid malignancy is distinct from genuine teratomas. The immunoprofile suggests primitive thyroid- or branchial cleft-like differentiation. Given that “blastoma” is a well-accepted terminology in the spectrum of DICER1-associated malignancies, the term “thyroblastoma” might be more convenient for these malignant teratoid tumors of the thyroid gland. Relationship of thyroblastoma to the DICER1 syndrome remains to be addressed.

**Electronic supplementary material:**

The online version of this article (10.1007/s00428-020-02853-1) contains supplementary material, which is available to authorized users.

## Introduction

Poorly differentiated malignancies of the thyroid gland are uncommon. They mainly encompass anaplastic thyroid carcinoma in the elderly and less frequently, poorly differentiated thyroid carcinoma. Primary teratomas of the thyroid gland are very rare neoplasms of presumable germ cell origin [1]. They represented < 0.1% (24/27,934) of all benign and malignant thyroid tumors at the former AFIP Institute [2]. In 2000, Thompson et al. reported a series of 30 primary thyroid teratomas and found some 250 case reports on cervical (including thyroid) teratomas in the literature prior to 2000 [2]. To date, ~ 300 cases have been reported in the literature, mainly as single case reports with rare series of up to 11 cases [3–5]. Thyroid teratomas present as large masses (up to 13 cm) with a mean tumor size of 6 cm [2, 3]. Clinically and histologically, thyroid teratomas are subgrouped into two categories: mature and immature teratomas and so-called malignant teratomas. Mature and immature teratomas recapitulate their gonadal and other extra-gonadal counterparts as they feature trilineage differentiation along the three germ layers but with highly variable proportions and degree of maturity [1–3]. Males and females are affected equally with considerable proportion of congenital and neonatal cases (mean age, < 10 years) [1, 2]. Half of the cases were histologically immature [1, 2]. Clinical outcome of mature/immature teratomas is determined mainly by age at presentation, tumor size, and extent of immature component but is generally excellent [2, 3].

So-called *malignant teratomas*, on the other hand, are much rarer and affect almost exclusively adults, with a highly aggressive clinical behavior leading to death of affected patients usually within first 2 years of diagnosis [2, 4]. Other than neuroectodermal overgrowth of the neuroblastemal component [2, 4], the presence of a heterologous (sarcomatous) mesenchymal component has not been emphasized in previous reports on *malignant thyroid teratomas* [2, 3]. We herein describe three cases of a highly aggressive thyroid malignancy combining teratoid epithelial component with extensive predominant heterologous (mainly rhabdomyoblastic) primitive mesenchymal overgrowth. While histogenetic relationship of these variants to immature teratomas and sarcomatoid yolk sac tumors is still not fully understood, current evidence suggests a distinct group of teratoid blastoma-like malignancies unrelated to genuine teratomas of the thyroid. We propose the term *thyroblastoma* to distinguish this highly lethal disease and separate it from conventional thyroid teratoma. If properly classified, the genetic background, possible heredity, biological properties, and therapeutic options of this poorly characterized aggressive malignancy would then be better addressed in the future.

## Materials and methods

Two cases (cases 1 and 3) were identified routinely and one (case 2) in the consultation files of the authors. Tissue specimens were formalin-fixed and processed routinely for histopathological evaluation. Immunohistochemistry (IHC) was performed on 3-μm sections cut from paraffin blocks using a fully automated system (“Benchmark XT System”, Ventana Medical Systems Inc., 1910 Innovation Park Drive, Tucson, AR, USA) and the following antibodies: thyroglobulin (Clone 2H11 + 6E1, RTU, Cell Marque, Rocklin, CA), calcitonin (Clone SP17, RTU, Cell Marque, Rocklin, CA), TTF1 (clone 8G7G3/1, dilution, 1:500, Zytomed Systems, Berlin, Germany), PAX8 (rabbit polyclonal, 1:50, Cell Marque), pankeratin (clone AE1/AE3, 1:40, Zytomed), p63 (clone SFI-6, 1:100, DCS), AFP (clone EP209, 1:150, Cell Marque), CD117 (clone EP10, 1:100, Quartett), beta-HCG (polyclonal, 1:3000, Dako), SALL4 (clone 6E3, 1:100, Zytomed), gylpican-3 (clone 1 g12, 1:200, Zytomed), OCT3/4 (clone N1NK, 1:100, Novocastra), D2–40 (clone D2–40, 1:50, Zytomed), PLAP (clone 8A9, 1:25, Dako), CD30 (clone Ber-H2, 1:40, Zytomed), NSE (clone BBS/NC/VI-H1, 1:300, Dako), CD56 (clone MRQ-42, 1:100, CELL MARQUE), TP53 (clone DO-7, 1:50, Dako), WT1 (clone 6F-H2, 1:50, Dako), WT1-c-terminus (polyclonal, 1:50, Santa Cruz), S100 (polyclonal, 1:2500, Dako), synaptophysin (clone SY38, 1:50, Dako), CD34 (clone BI-3C5, 1:200, Zytomed), desmin (clone D33, 1:250, Dako), myogenin (clone F5D, 1:50, Dako), CDX2 (clone CDX2–88, 1:100, DCS), CK20 (clone Ks20.8, 1:50, Dako), HepPar-1 (clone OCH1E5, 1:200, Dako), NUT (clone C52B1, 1:45, Cell Signaling), TLE1 (polyclonal, 1:200, Santa Cruz), SMARCB1/INI1 (clone MRQ-27, dilution, 1:50, Zytomed), SMARCA4 (anti-BRG1 antibody, clone EPNCIR111A, 1:100, Abcam; Cambridge, UK), Ki-67 (clone 30–9, RTU, Ventana, Tucson, Arizona), chromogranin A (Clone LK2H10, 1/300, Cell Marque, Rocklin, CA), CD99 (Clone O13, RTU, Cell Marque, Rocklin, CA), GFAP (Clone GFA, 1/1000, DakoPatts, Denmark), and HMB45 (Clone HMB-45, 1/300, Cell Marque, Rocklin, CA).

### Molecular testing

After careful manual microdissection, DNA was analyzed from FFPE tumor tissue using the Maxwell© 16 system (Promega, Madison, WI, USA) according to manufacturer’s instructions. *DICER1* sequence analysis was performed using the QIAseq Targeted Human Comprehensive Cancer Panel according to manufacturer’s instructions (the list of the 160 genes is shown in the supplementary file). Bioinformatic evaluation of the sequencing data, including variant calling and annotation, was done with the CLC Genomics Workbench (QIAGEN, Redwood City, CA, USA). Low-quality variants with a score under 200 were filtered out, as well as variants in non-protein-coding regions, synonymous variants, and those present in GnomAD with an allele frequency of over 1%. The remaining variants were assessed for pathogenicity according to ACMG/AMP criteria. The *DICER1* variants were classified as described previously [6].

## Case histories

### Case 1

A 17-year-old male presented with a recent history of a rapidly growing mid-cervical mass. Imaging confirmed the presence of a mass diffusely infiltrating both thyroid lobes, mainly the right lobe measuring 8.2 cm. Following frozen section examination, which suggested a malignant germ cell neoplasm, subtotal thyroidectomy was performed with involved margins. This was followed by two cycles of germ cell neoplasm-directed chemotherapy (cisplatin, etoposide and ifosfamide, PEI) and then re-excision of the tumor bed. Postoperative chemotherapy was continued with an additional 4 PEI cycles simultaneous to local radiotherapy. The patient is alive under ongoing chemotherapy (three consolidating soft tissue sarcoma cycles; 2x I^2^VAd, 1xI^2^VA) 8 months after initial diagnosis.

### Case 2

The patient is a 17-year-old euthyroid female with a rapidly growing, large mass in the thyroid with high values of anti-thyroglobulin antibodies (196.5 (0–115) UI/ml) and anti-microsomal antibodies (1279.5 (0–35) UI/ml). The ultrasound examination documented a 6.3-cm mass involving both thyroid lobes and isthmus. Preoperative fine-needle aspiration cytology was interpreted as suspicious for medullary carcinoma. The patient underwent total thyroidectomy. The patient received two cycles of chemotherapy. The first of 3 days: Etoposide (150 mg/m2) 250 mg, vincristine 2 mg (first day), ifosfamide 2000 mg, and actinomycin (0.5 mg/m2) 0.8 mg. The second of 4 days, approximately 1 month later: Beomycin 30 ng (first day), etoposide 200 mg, and cisplatin 45 mg. The disease rapidly progressed into the mediastinum even after chemotherapy, and the patient died of the disease 1 year after the diagnosis.

### Case 3

A 45-year-old woman with a history of multinodular goiter (verified histologically as benign 3 years ago) was admitted because of recent onset of dysphasia and painful rapid increase in thyroid size over the previous 3 months. Clinical examination showed an enlarged firm right thyroid lobe with no lymphadenopathy. Laboratory investigations were normal. Neck CT showed a 6.1 × 3.8 × 3.7 cm right lobe mass with retrosternal extension. No other manifestations were seen on thoracic and abdominal CT. FNA showed an unclassified high grade sarcomatous neoplasm. The patient received a total thyroidectomy and recovered well postoperatively. Follow-up was not available.

## Pathological findings

### Case 1

**Fig. 1:**
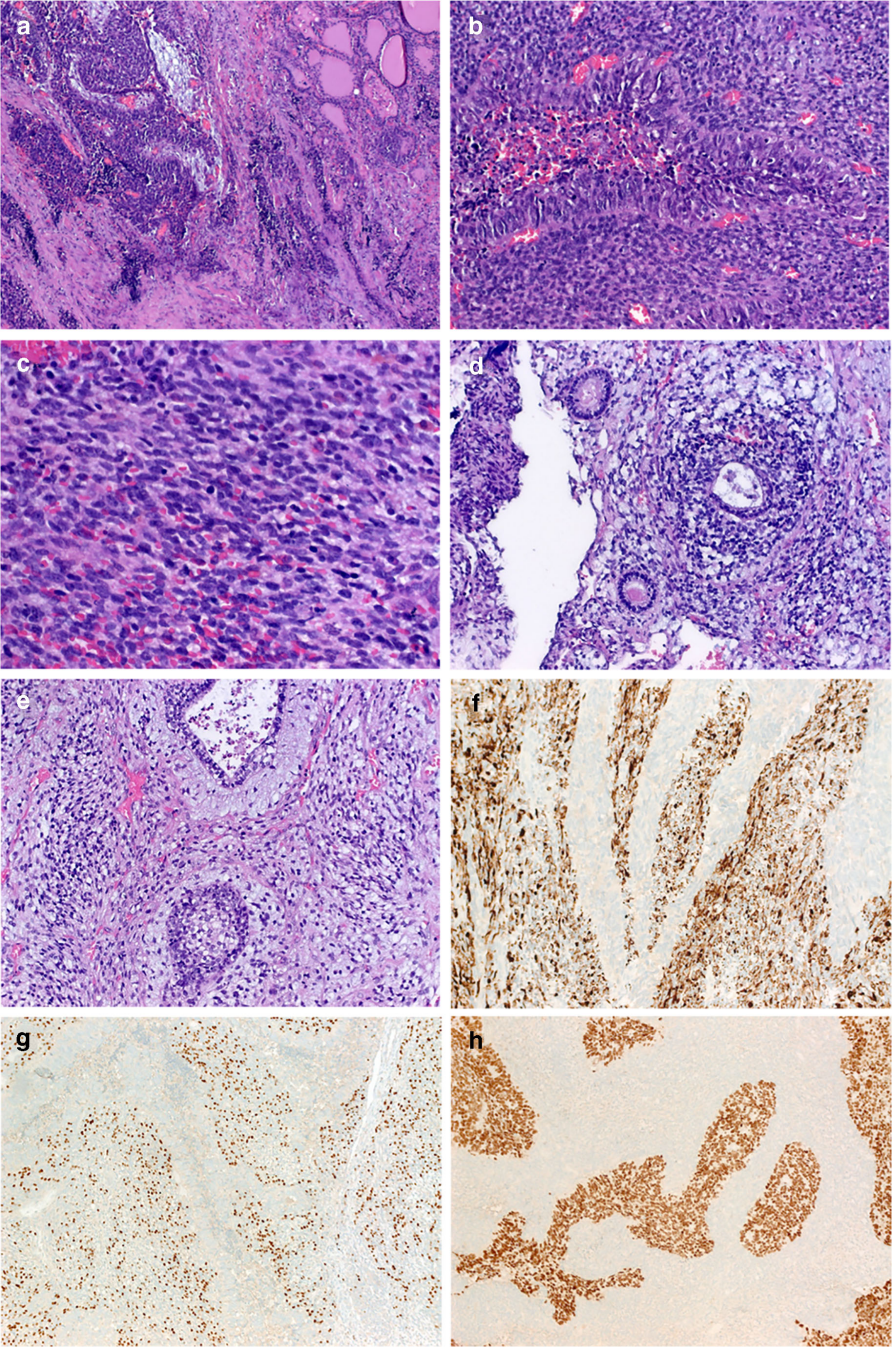
Representative images of case 1. **a** Highly infiltrative growth amid thyroid tissue. Most areas show biphasic growth with cohesive basophilic large columnar cells surrounded by cellular mesenchymal-type stroma (**b**; higher magnification of spindled stroma in **c**). **d** _Primitive intestinal-type and respiratory-type tubules are seen, focally encased by primitive small cell stroma. **e** Tubules and clear cell squamoid nests are surrounded by neuroepithelial-type matrix. The stromal component strongly expressed desmin (**f**) and myogenin (**g**). SALL4 was limited to the cohesive epithelial-like component (**h**)

The surgical specimen consisted of right thyroid lobe of 4.8 × 3.2 × 2.5 cm and the left lobe of 4.8 × 3.8 × 1.8 cm. On cut-surface, a gray-whitish to tan-yellow extensively necrotic mass was seen replacing most of the right lobe and extending variably into the left lobe (Fig. 1a). Areas of hemorrhages and necrosis were prominent.

Histologically, the tumor was triphasic, composed mainly of primitive small round to oval cells, disposed into variably sized and shaped, occasionally communicating, compact aggregates with foci of necrosis and brisk mitotic activity. The periphery showed frequent palisading of the nuclei (Fig. 1b). This cohesive component was surrounded by a variably cellular primitive component of mainly spindled cells arranged into elongated fascicles (Fig. 1c). Within these areas and between cellular tumorous nodules were scattered epithelial structures in different proportions surrounded by primitive variably myxoid connective tissue stromal elements (Fig. 1d). Apoptotic figures were abundant, as well as mitoses (> 20 mitoses per 10 HPF). The epithelial elements were mainly tubular glands lined by fetal-type vacuolated columnar epithelium with a variable rosette-like appearance and periglandular cuffs of primitive stromal cells (Fig. 1d). Small aggregates of fetal-type clear cell squamous epithelium were seen (Fig. 1e). No cartilage, pilosebaceous elements, other skin adnexal structures, or mature adult-type organoid tissues were seen. Immature neuroepithelium was present focally (Fig. 1e), but a malignant conventional germ cell component was not detected. By IHC, the stromal component showed diffuse expression of desmin (Fig. 1f) and myogenin (Fig. 1g) but only very limited focal cytokeratin reactivity. The small cell component expressed SALL4 diffusely (Fig. 1h) and glypican-3 and synaptophysin focally but was negative with all other markers. TP53 IHC showed very strong mutation-type reactivity in the compact small cell component but a wild-type pattern in the mesenchymal stromal component. Pankeratin, TTF1, PAX8, and variably CD56 were expressed in the scattered tubules. Neuroepithelial-like foci expressed NSE and synaptophysin. All other markers listed above in the method section including germ cell markers, thyroglobulin, calcitonin, chromogranin A, cytokeratin 20, CEA, p63, CD99, NUT, HMB45, S100 protein, GFAP, and neurofilament were negative. Nuclear SMARCA4 expression was retained in the different tumor components. The surrounding (residual) thyroid tissue showed no evidence of goiter.

### Case 2

**Fig. 2:**
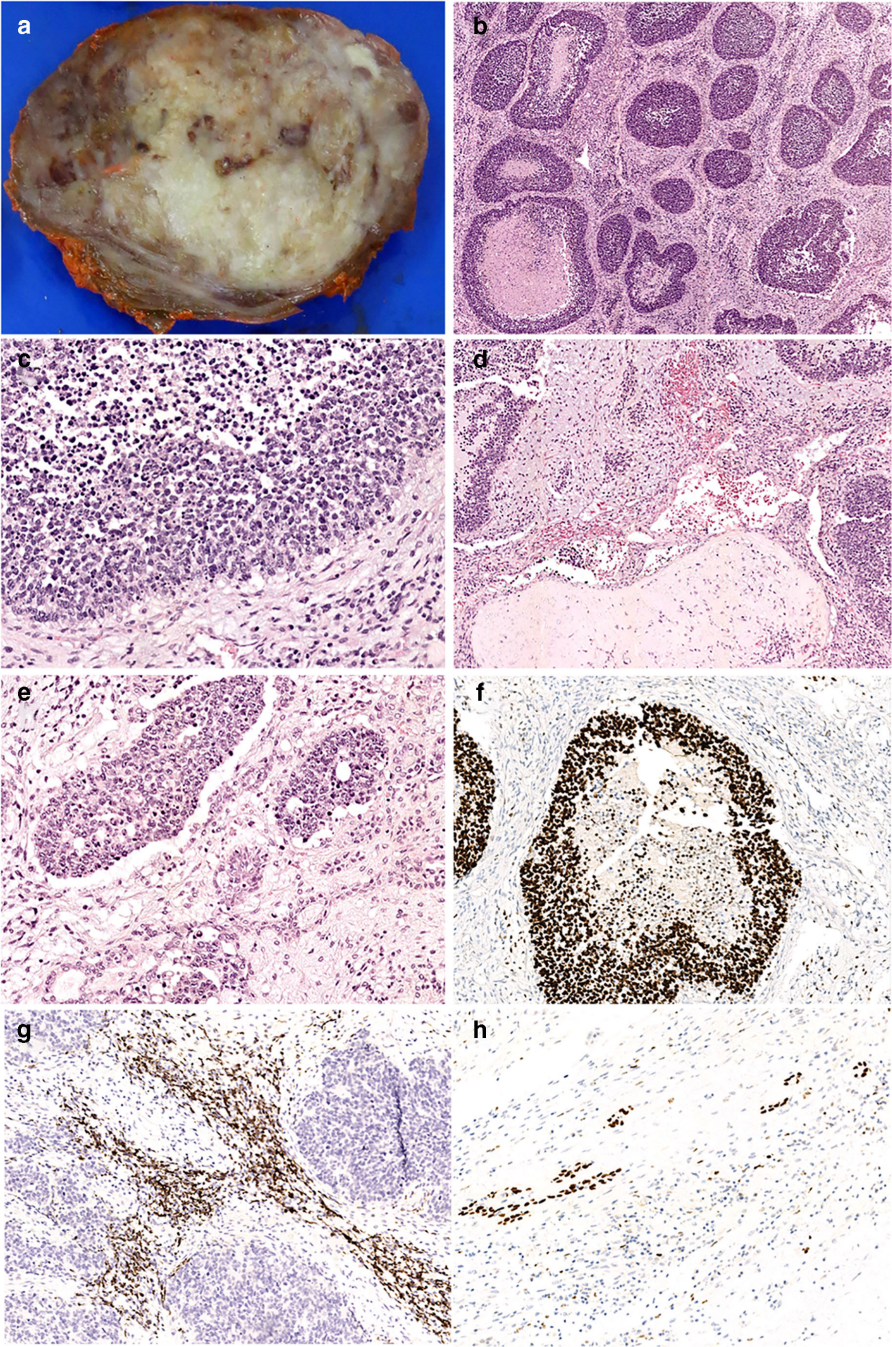
Representative images of case 2. **a** Macroscopic aspect of the tumor. **b** HE, nested pattern with necrosis and rich stromal component. **c** HE, small cell component with apoptotic and mitotic figures. **d** HE, chondroid matrix and cellular stroma. **e** HE, small cell (top) and epitelial tubular component (bottom). **f** SALL4 in the small cell component. **g** Desmin in the stromal component. **h** p63 in the epitelial component

The surgical specimen disclosed a whitish and heterogeneous 6-cm nodule with infiltrative margins on cut surface (Fig. 2a). Histologically, the nodule corresponded to an infiltrative neoplasm with invasion of the perithyroidal adipose tissue and vascular invasion. The neoplasm had 3 components: a small cell undifferentiated/immature component (Fig. 2b, c), a tubular component, and a stromal component (Fig. 2d, e). The small cell component was composed of cells with scant cytoplasm and round to oval nuclei, arranged in nest with comedo-type necrosis (Fig. 2b, c). Apoptotic figures were abundant, as well as mitoses (>20 mitoses per 10 HPF), including atypical mitoses. The tubular component was dispersed throughout the neoplasm and was composed of tubular structures with one or two layers of bland-looking cuboidal cells. The stromal component was exuberant and contained immature spindle cells, as well as mature and immature cartilage nests (Fig. 2d).

The small cell component expressed TTF1, NSE, glypican3, SALL4 (Fig. 2f), and Ki-67 in > 90% of the cells. Expression of thyroglobulin, calcitonin, chromogranin A, synaptophysin, AE1AE3, cytokeratins 8/18, cytokeratin 20, CEA, p63, CD99, NUT, HMB45, desmin, S100 protein, GFAP, neurofilament, CD30, and CD45 were not detected in the small cells. The stromal component expressed desmin (Fig. 2g), myogenin, S100 protein, CD99, and focally p63. The tubular component expressed AE1/AE3, cytokeratins 8/18, p63 (Fig. 2h), and TTF1. EWSR1 rearrangements were not detected. Nuclear SMARCA4 expression was retained in the different tumor components. Figure 2 illustrates representative examples of the histological and immunohistochemical features of this case. Nuclear SMARCA4 expression was retained in the different tumor components. The surrounding (residual) thyroid tissue showed no evidence of goiter.

### Case 3

**Fig. 3:**
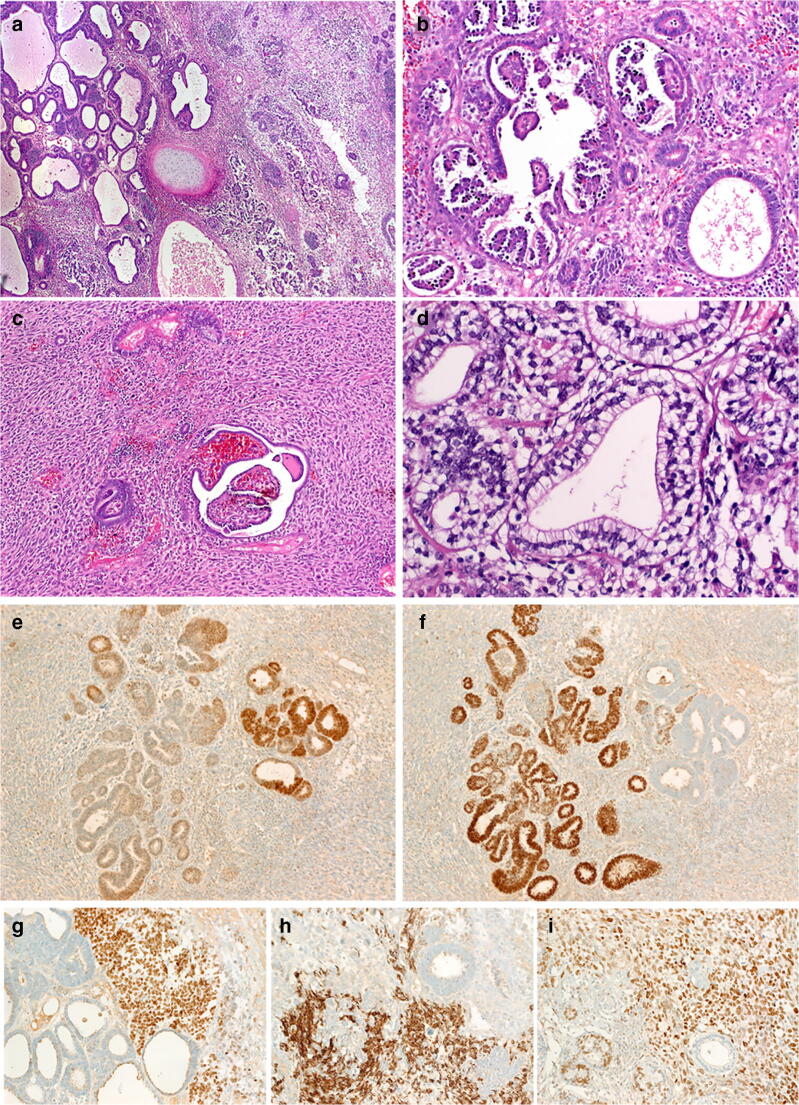
Representative images of case 3. **a** Biphasic (right) and epithelial/tubule-predominant areas were seen juxtaposed in this area, note centrally located cartilage island. **b** The epithelial component was composed of primitive tubules lined by basophilic columnar cells admixed with glomeruloid papillary structures. **c** Highly cellular sarcomatoid stroma with scattered intestinal-type tubule and glomeruloid structures are seen. **d** In some areas, fetal-type tubules lined by clear cells are evident. Expression of PAX8 (**e**) and TTF1 (**f**) is predominantly mutually exclusive. Primitive small cell stroma shows variable expression of TTF1 as well (**g**). Otherwise, the stroma was focally desmin-positive (**h**) and diffusely SALL4 positive (**i**), note that tubules inconsistently expressed SALL4 in **i**

This case was also composed of three components but differed in some aspects (Fig. 3a, b, c). The mesenchymal component comprised > 50% of the tumor and corresponded to large cell pleomorphic undifferentiated sarcomatous malignancy similar to the so-called MFH of the soft tissue (“UPS”) and contained focal areas with bizarre multinucleated cells. The epithelial component was either in the form of scattered glands and glomeruloid structures or was represented by focally confluent predominantly epithelial areas composed of tubules, solid aggregates, and glomeruloid structures similar to epithelial Wilms tumor of the kidney (Fig. 3a, b, c). Primitive tubular glands and neuroepithelial rosette-like glands were seen as well. Microscopic foci of mature and immature cartilage were seen (Fig. 3a), but none contained mature organoid adult-type structures, pilosebaceous or skin adnexal structures. Focal fetal-type tubules lined by clear cells were seen (Fig. 3d). The epithelial component co-expressed TTF1 and PAX8, suggesting a thyroid-like line of differentiation. Notably, PAX8 (Fig. 3e) and TTF1 (Fig. 3f) showed an inverse reactivity to each other, although a subset of cells seems to co-express both to a variable extent. The primitive stromal component amid the epithelial glands expressed TTF1 variably (Fig. 3g), desmin (Fig. 3h), and myogenin. SALL4 was strongly but variably expressed in both the epithelial and the sarcomatous component (Fig. 3i). All other specific germ cell markers (OCT-3/4, beta-HCG, AFP, PLAP, CD30 and D2–40) as well as the lineage-specific markers listed above were negative. Nuclear SMARCA4 expression was retained in the different tumor components.

## Molecular results

Sufficient tumor tissue was available for molecular testing in case 1 and case 2. In case 1, a known *DICER1* “hotspot” somatic missense mutation (p.Asp1709Asn; c.5125G > A) was found. This mutation occurs in an exon that encodes part of the RNAse IIIB domain, which is critical for the correct cleavage of hairpin precursor microRNAs to their mature products [7, 8]. Case 2 was also found to possess a known pathogenic missense mutation in *DICER1* p.Gly1809Arg. This is an established somatic mutation, in same RNAse IIIB domain as p.Asp1709Asn. In both cases, the variant allele frequency was consistent with these variants being present in the heterozygous state (Table 1). There was no evidence of a “second hit” in *DICER1* in either tumor. Other variants likely to be associated with thyroid cancer were not found. In addition to these *DICER1* variants, a pathogenic variant in *TP53* (c.400 T>; p.Phe134Leu) was found in case 1, consistent with the mutation-type reactivity seen on IHC in this tumor. This variant appears to be present at a heterozygote allele frequency.

**Table 1:** Clinicopathological and molecular features of reported DICER1-related teratoid thyroid malignancies including current study (*n* = 8)

No	Author/s	Age/ Gender	Size cm	Presentation	Treatment	Follow-up	Teratoid epithelial component	Stromal pattern	Cartilage	Family history of thyroid or other DICER1-related neoplasms	Pathogenic *DICER1* variants* (variant allele fraction)
1	Rabinowits et al. [9]	59/F	6.7	Rapidly growing neck mass, hoarseness	Surgery, CT	6 mo after diagnosis, CT and surgery for residual disease performed, no extended FU	Primitive microfollicular tubules TTF1	Primitive PNET-like	NA	NA	p.E1813G (0.46)
2	Yang et al. [10]	45/F	2.8	Neck mass	Surgery, CRT	Lung mets (7 mo), DOD (11 mo)	Primitive microfollicular tubules TTF1+, PAX8+	Primitive rhabdomyoblastic	Absent	No	p. E1705K
3	Rooper et al. [11]	65/F	1.9	Neck mass	Surgery, CT	NED (125 mo)	Neuroepithelial, primitive microfollicular tubules TTF1+, PAX8+	Primitive rhabdomyoblastic	Present	No	p.E1705K (0.43); p.Y819fs (0.47)
4	Rooper et al. [11]	29/F	10	Neck mass	Surgery, CRT	DOD (53 mo)	Neuroepithelial, primitive microfollicular tubules TTF1+, PAX8+	Primitive rhabdomyoblastic	Absent	No	p.E1813G (0.33); p.V448fs (0.40)
5	Rooper et al. [11]	42/F	8	Neck mass	Surgery, CRT	NED (64 mo)	Neuroepithelial, primitive microfollicular tubules TTF1+, PAX8+	Primitive rhabdomyoblastic	Present	No	p.E1813Q (0.46); p.K868Ter (0.48)
6	Rooper et al. [11]	60/M	1.7	Incidental on imaging	Surgery	DOD (10 mo)	Neuroepithelial, primitive microfollicular tubules TTF1+, PAX8+	Primitive rhabdomyoblastic	Present	No	p.D1810H (0.09)
7	Current	17/M	8.2	Rapidly growing neck mass	Surgery, CT	NED (8 mo)	Respiratory, enteric & neuroepithelial tubules, Neuroepithelial, primitive microfollicular tubules TTF1+, PAX8+	Primitive rhabdomyoblastic	Absent	No	p.D1709N (0.62).
8	Current	17/F	6.3	Rapidly growing neck mass	Surgery, CT	DOD (12 mo)	Respiratory, enteric & neuroepithelial tubules, primitive microfollicular tubules TTF1+, PAX8+	Primitive, focal rhabdomyoblastic	Present	No	p.G1809R (0.59)

*CRT*, chemoradiotherapy; *CT*, chemotherapy; *DOD*, died of disease; *F*, female; *FU*, follow-up; *M*, male; *mets*; metastases; *mo*, month; *NA*, not available; *NED*, no evidence of disease

*Variants are reported as protein changes. p. E1705K, p.D1709N, p.G1809R, p.D1810H, p.E1813G, and p.E1813Q are all single amino acid changes at hotspot residues directly (E1705, D1709, D1810, E1813) or indirectly (G1809) interact with magnesium or manganese ions to facilitate enzymatic cleavage of precursor microRNA hairpins to 3′ and 5′ mature miRNA products. The variant amino acids result in improper cleavage. The VAFs vary from 0.09 to 0.62. They are almost certainly somatic in origin. p.Y819fs, p.V448fs, and p.K868Ter are all predicted to truncate the DICER1 protein. The VAFs are all between 0.40 and 0.50, and therefore, a germline origin for these variants is possible. For cases 3, 4, and 5, the tumors contain two DICER1 variants—one hotspot variant and one variant that is predicted to truncate the protein. Previous work has established that this combination of variants nearly always occurs in trans (i.e., they are biallelic). The other cases report only variant (hotspot in all cases), but in the absence of a complete evaluation of the *DICER1* locus, including expression studies, it is not possible to conclude that these single “hits” are unaccompanied by other DICER1 variants, usually in trans (as discussed above). For more details, see de Kock et al., 2019 [8]

## Discussion

The tumor type we are describing herein has been likely included in the spectrum of what has been named “malignant thyroid teratoma” in the past. Although this term has been largely limited to a subset of thyroid gland malignancies believed to be of germ cell origin, there has been no convincing rational to distinguish immature from malignant teratoma on the basis of morphology alone. Moreover, the terms “immature” and “malignant” are not established histological categories in the pathology of gonadal germ cell neoplasms. Hence, distinction of *immature* from so-called malignant thyroid teratoma has relied mainly on the distinctive demographic and prognostic differences between the two disease categories. Immature thyroid teratomas are essentially neonatal or pediatric diseases with excellent outcome after complete surgical removal [2, 3]. On the contrary, so-called malignant teratoma is a disease of adults and the elderly with a mean age at diagnosis of > 40 years. At same time, this variant is highly aggressive with the majority of affected patients succumbing to their disease sooner after diagnosis or treatment trials [2, 3, 4, 5]. In line with their germ cell origin, genuine mature/immature thyroid teratomas frequently contain tissue derivatives from all three germ cell layers, including, in particular, different types of mature organoid adult-type epithelia admixed with pilosebaceous units and other skin adnexal structures [2, 3]. Immature neuroblastemal tissue elements are seen in immature cases [2].

The current cases, however, display many significant differences from the reported malignant thyroid teratomas, suggesting it might represent a different entity. In particular, reported thyroid teratomas only rarely show overt sarcomatous (mainly primitive rhabdomyoblastic) stromal overgrowth, similar to our cases [12]. Myo-D1 is expressed however in immature mesenchymal areas of some teratomas, indicating early skeletal muscle differentiation [2]. On the other hand, mature and immature tissue derivatives of neuroectodermal origin as a hallmark feature in the majority of mature and immature teratomas were either lacking or limited in our cases. Likewise, the absence of a conventional germ cell component and the expression of classical germ cell markers other than SALL4 are strong arguments against a true germ cell origin.

In line with a distinct clinicopathological and molecular entity, malignant teratomas lack isochromosome 12, a genetic hallmark in the majority of germ cell neoplasms [9, 13–16]. Rabinowits et al. reported in 2017 for the first time the presence of a pathogenic *DICER1* mutation (c.5438A > G; p.Gln1813Glu) in a case of malignant thyroid teratoma in a 59-year-old female [9]. The tumor revealed a primitive neuroectodermal tumor (PNET)-like transformation. The authors linked the *DICER1* mutation to the PNET-like transformation [9]. Sequencing of paired tumor and normal tissue samples indicated a somatic nature of the detected *DICER1* mutation [9]. During preparation of this study, another paper was published by Rooper et al. describing pathogenic *DICER1* mutations in 4 of 4 malignant but in none of 4 mature/immature thyroid teratomas [11]. The age of onset (29 to 65 years) and negative family history suggested that the *DICER1* variants identified were somatic in nature [11] and they stay in sharp contrast to those inherited DICER1-related neoplasms [17, 18]. Our current study confirmed the presence of pathogenic *DICER1* missense mutations, occurring in exons encoding the critical RNase IIIB domain of DICER1 in two tumors. No second hits were seen, and the allele frequency of the variants is consistent with retention of heterozygosity.

The question may be raised as to whether our current cases (in particular case 3) might represent genuine carcinosarcomas. Indeed, case 3 has been originally diagnosed as such. In the current WHO classification [19], carcinosarcoma is considered a morphological pattern in the spectrum of anaplastic carcinoma and not as a distinctive entity. To date, some 30 cases of *thyroid carcinosarcoma* have been reported [10, 20]. However, the term carcinosarcoma has been used inconsistently for neoplasms combining a differentiated conventional (mostly follicular or papillary) carcinoma component and a sarcomatoid component. Accordingly, it is likely that some if not the majority of those reported carcinosarcoma cases represented dedifferentiated follicular carcinomas or anaplastic carcinoma variants [19, 20]. More importantly, the carcinosarcoma case reported by Yang et al. affected a 45-year-old female, showed similar teratoid glands as in our cases, and revealed a *DICER1* mutation in the tumor, confirming similarity to our cases and to the cases reported by Rooper et al., both histologically and genetically [10, 11]. Although we could not obtain molecular findings for our case 3, this case was very similar to the DICER1-mutated case reported by Yang et al. (both affected females aged 45), suggesting that also case 3 belongs to the same disease spectrum as cases 1 and 2. Moreover, our three cases are distinct from poorly differentiated thyroid carcinoma of childhood and adolescence, a recently reported entity characterized by *DICER1* mutations as well [21]. This poorly differentiated thyroid carcinoma variant does not contain teratoid or heterologous mesenchymal components [21].

Spindle epithelial tumor with thymus-like elements (SETTLE) is another mixed epithelial and stromal thyroid neoplasm with presumed branchial cleft-like differentiation [22]. Indeed, the terms “thymoblastoma or thyroblastoma” were discussed as possible explanation for the varied histology of SETTLE [22, 23]. However, the prominent teratoid pattern, the uniformly high-grade morphology with brisk mitotic activity, necrosis, pleomorphism and other frankly malignant features, and the uniformly highly aggressive clinical course are not features of SETTLE [24]. A recent NGS study did not show any *DICER1* mutations or consistent molecular findings in SETTLE [25].

Due to its favorable prognosis, the rare entity “carcinoma of the thyroid with Ewing family tumor elements (CEFTE)”, also called adamantinoma-like Ewing family tumor, should be recognized and distinguished from thyroblastoma and other aggressive thyroid malignancies with monomorphic small basaloid cells [26, 27]. At variance with the cases reported herein, CEFTE expresses consistently p63 and CD99 and harbors the typical *EWSR1/FLI1* rearrangement [28].

The DICER1 syndrome represents an emerging inherited multineoplastic disorder caused by germline *DICER1* gene mutations and characterized by an array of topographically and phenotypically diverse neoplasms of benign, low-grade, or aggressive nature [8, 17, 18]. Common to these neoplastic lesions is the presence of hamartoma-like or teratoma-like admixture of diverse tissue derivatives, frequently with a benign-looking organotypical epithelial component such as seen in Müllerian adenosarcoma, cervical embryonal rhabdomyosarcoma, and intracranial sarcomas [8, 17, 18]. Notably, many of these DICER1-associated neoplasms have some site dependent morphological resemblance to the developmental stages in organogenesis, resulting into a teratoid or blastomatous appearance in many of them. Foci of cartilage are another common feature of several DICER1-associated lesions and represent a strong histological clue to suspicion of the disorder [29]. Indeed, the presence of cartilage in these DICER1-related teratoid thyroid neoplasms might have enhanced misinterpretation of malignant thyroid teratoma as being related to genuine germ cell tumors. In this context, it is worth mentioning that *DICER1* mutations are very uncommon in germ cell neoplasms [30, 31].

The list of organ manifestations of the DICER1 syndromes are growing steadily and encompass sinonasal (chondromyxoid hamartomas), thyroid (multinodular goiter, poorly differentiated carcinomas), gonadal (sex cord stromal tumors), genital (cervical embryonal rhabdomyosarcoma, Müllerian adenosarcoma), renal (cystic nephroma and anaplastic sarcoma of kidney), thoracic (pleuropulmonary blastoma), intracranial (pituitary blastoma, pineoblastoma, PNET, sarcomas), and others [8, 17, 18, 21, 32].

Recently, malignant teratoid sacrococcygeal tumors occurring in two infants and harboring pathogenic germline *DICER1* mutations were reported [33]. The histology is highly reminiscent of the cases we are describing herein and is similar to those reported by Rooper et al. with a combination of immature teratoma-like and rhabdomyosarcoma-like areas and foci of cartilage as well [11, 33]. Additional *DICER1*-associated neoplasms were diagnosed in one of the two children and a presumed intracranial metastasis in the other [33]. Genetic analysis revealed biallelic pathogenic germline *DICER1* mutations in both. The authors discussed the probability these teratoma-like lesions being a novel DICER1-related entity.

A last molecular pathogenetic point to address is the striking similarity of the tumor we are reporting to sinonasal teratocarcinosarcoma, a similarly aggressive primitive multiphenotypic malignancy reported initially by Heffner and Hyams as “malignant teratoma” and affecting predominantly adults at a mean age of 60 years [34]. The molecular pathogenesis of this tumor remained elusive until our group recently identified recurrent SMARCA4 loss as driver genetic event in the majority of cases [35]. To verify any potential relationship between the two entities, we tested our current cases for SMARCA4 expression; all showed retained nuclear reactivity, excluding molecular relationship to sinonasal-type teratocarcinosarcoma. Notably, a distinctive infantile pulmonary teratoid tumor has been reported which harbored biallelic *SMARCA4* mutations [36]. Taken together, these very recent studies highlight the existence of two distinctive categories of aggressive malignant teratoid tumors unrelated to genuine germ cell neoplasms: one driven by *SMARCA4* inactivation and another related to *DICER1* mutations.

Based on the above observations, we believe that the current cases and possibly the majority of what has been called malignant thyroid teratomas in the past are probably distinctive DICER1-related primitive malignant teratoid thyroid tumors that are distinct from genuine mature and immature thyroid teratomas. The frequent presence of TTF1+/PAX8+ follicle-like structures indicates organotypical differentiation or primitive thyroid-like elements. This observation is in line with several DICER1-related primitive neoplasms that recapitulate the organ of origin, a finding reflected in the predominance of the “blastoma” terminologies (pleuropulmonary blastoma, pineoblastoma, pituitary blastoma, and others) for several DICER1-related malignancies [17, 18, 32]. Thus, in analogy to these many DICER1-associated “organ blastomas,” we propose the term “thyroblastoma” for the neoplasm under consideration. We believe that these cases represent another novel subtype of DICER1-associated tumors, irrespective of being of sporadic or germline origin.

Looking at the 8 *DICER1*-mutated “thyroblastoma” cases reported to date (Table 1), there is a striking predilection for females (6 of 8) with an age range of 17 to 65 years (median, 43). Given that one previous case was reported as carcinosarcoma and one of our current cases (although without molecular testing) was initially diagnosed as such, it is likely that this entity is under-recognized and hides behind so-called malignant teratomas, carcinosarcomas, or SETTLE. They have in common a triphasic pattern composed of (1) TTF1+/PAX8+ primitive teratoid follicle-like glands admixed with neuroepithelial-like and fetal tubule-like elements, (2) primitive small cell component, and (3) variably cellular mesenchymal stroma with frequent rhabdomyoblastic differentiation. Foci of cartilage are common (4 of 7 cases). Follow-up was available for 7 patients (range, 8–128 months; median, 12). Four patients died of disease at 10–53 months (median, 11.5). This underlines the almost invariably highly aggressive course of thyroblastoma, in contrast to the low malignant potential of some other organ blastomas. Higher age at presentation and lack of personal or family history of other neoplasms all argue for a sporadic neoplasm unrelated to the inherited DICER1 syndrome. Recognizing this variant, for which we propose the term “thyroblastoma,” and distinguishing it from immature thyroid teratoma is mandatory to better delineate its clinicopathological spectrum and critically assess its possible association with the DICER1 syndrome.

## Electronic supplementary material

ESM 1 (XLSX 9 kb)
